# Development and Performance Analysis of an Automatic Core Cutter for Elephant Apple (*Dillenia indica* L.) Processing

**DOI:** 10.3390/foods13060848

**Published:** 2024-03-11

**Authors:** Deepanka Saikia, Radhakrishnan Kesavan, Minaxi Sharma, Baskaran Stephen Inbaraj, Prakash Kumar Nayak, Kandi Sridhar

**Affiliations:** 1 Department of Agricultural Engineering, Centurion University of Technology and Management, Paralakhemundi 761211, India; deepankasaikia@gmail.com; 2 Department of Food Engineering and Technology, Central Institute of Technology Kokrajhar, Kokrajhar 783370, India; 3 Department of Applied Biology, University of Science and Technology Meghalaya, Baridua 793101, India; 4 Department of Food Science, Fu Jen Catholic University, New Taipei City 242062, Taiwan; sinbaraj@yahoo.com; 5 Department of Food Technology, Karpagam Academy of Higher Education (Deemed to be University), Coimbatore 641021, India

**Keywords:** underutilized fruit, postharvest processing, machine design, automatic core cutter, cutter efficiency, cost analysis

## Abstract

Elephant apple, a fruit with numerous bioactive compounds, is rich in therapeutic qualities. However, its use in processed products is limited due to insufficient postharvest processing methods. To address this issue, an automatic core cutter (ACC) was developed to handle the hard nature of the fruit while cutting. The physical characteristics of the elephant apple were considered for designing and development of the cutter. The cutter is divided into four main sections, including a frame, collecting tray, movable coring unit, and cutting base with five fruit holders. The parts that directly contact the fruit are made of food-grade stainless steel. The efficiency of the cutter was analyzed based on cutting/coring capacity, machine efficiency, loss percentage, and other factors, and was compared to traditional cutting methods (TCM) and a foot-operated core cutter (FOCC). The ACC had an average cutting/coring capacity of 270–300 kg/h, which was significantly higher than TCM’s capacity of 12–15 kg/h and comparable to FOCC’s capacity of 115–130 kg/h. The ACC offered a higher sepal yield of 85.68 ± 1.80% compared to TCM’s yield of 65.76 ± 1.35%, which was equivalent to the yield obtained by FOCC. Therefore, the ACC outperforms TCM in terms of quality, quantity, and stress associated and is superior to FOCC in terms of higher efficiency of machine and labor.

## 1. Introduction

**Figure 1:**
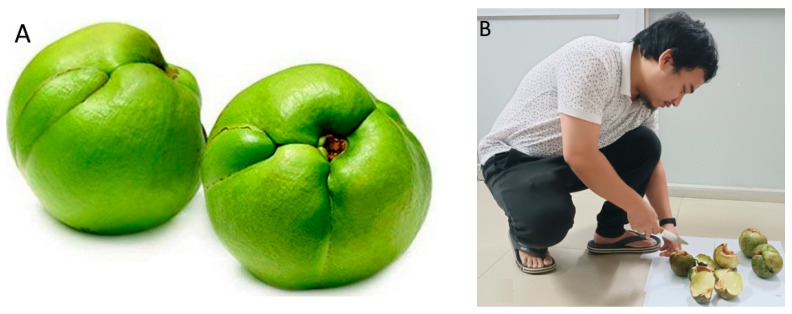
Visual observation of (**A**) elephant apple and (**B**) traditional method of cutting.

The northeast region of India is rich in native plant products; however, the use of these agricultural produce is limited due to inadequate postharvest processing techniques. *Dillenia indica*, commonly known as elephant apple (Figure 1A), is an underutilized agricultural fruit crop found in several states of India, as well as in other countries [1]. Elephant apple fruit has a wide range of therapeutic benefits, including anti-oxidant, anti-microbial, anti-cancer, anti-diabetic, anti-diarrhoeal, anti-mutagenic, and immunostimulating activities [2,3,4,5,6,7,8,9].

Elephant apple cutting is a laborious and time-consuming technique, making it less utilized on a large commercial scale. The five closely adhering imbricate sepals of the hard, densely coated fruit need to be separated one by one, which increases the time, labor, and expense. Elephant apples have traditionally been cut by hand using a regular knife (Figure 1B), and the process takes 105–115 s per fruit, based on the individual’s skill. Subsequently, improper cutting of the fruit can also cause problems during later processing phases, and cutting the hard fruit with a knife puts the person at risk of getting hurt. Therefore, it is important to appropriately remove the central part of the fruit with the least amount of wastage. Also, an individual’s capacity to cut a large quantity of fruits is influenced due to the inefficiency and time requirement involved in the traditional cutting method (TCM) [1].

**Figure 2:**
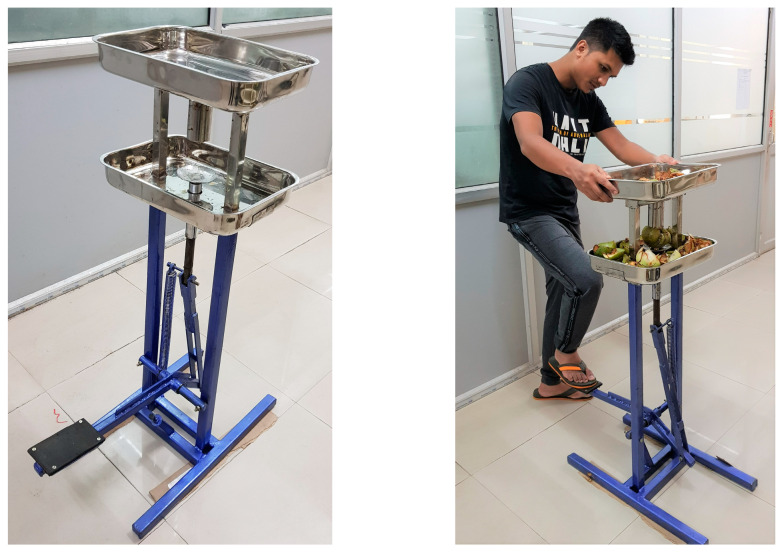
Foot-operated core cutter (FOCC). This figure is reproduced from Saikia et al. [1] and is an open access article (copyright © 2024 by authors under exclusive license to Springer Nature Switzerland AG) distributed under the terms and conditions of the Creative Commons Attribution (CC BY) license.

There is limited literature available on the development of an automatic core cutter (ACC) for elephant apple processing. However, a study on a foot-operated core cutter (FOCC) can be used as a reference for the development of an ACC for elephant apple processing. A study by Saikia et al. [1] developed a FOCC (Figure 2) with connected shafts for fruit cutting, adjustable core cutter, and separate collecting boxes for sepals and cores, optimizing fruit processing efficiency. The FOCC demonstrates a 12.24% improvement in processing time compared to TCM and a 133.2% increase in sepal yield. This study demonstrated that a FOCC can significantly reduce the processing time and increase the efficiency of the process. Therefore, it is feasible to develop an ACC for elephant apple processing to decrease processing time, minimize human effort, and enhance production yield.

This study aims to determine the physical characteristics of the elephant apple fruit and to develop an ACC. Moreover, the efficiency of the ACC in terms of cutting/coring capacity, machine efficiency, loss percentage, and other factors were compared with TCM and FOCC.

## 2. Materials and Methods

### 2.1. Collection of Elephant Apple Fruit

Elephant apple fruits (100) in matured and damaged-free form have been obtained from the local market of Kokrajhar, Assam, India. Before the experimental trials, the fresh fruits were washed and cleaned for any foreign matters and stored at 25–30 °C for 4 h in a stable environment. The fruits were divided into two groups initially, group 1 and group 2, where group 1 included larger-sized fruits (50), and group 2 included smaller-sized fruits (50). Visual inspection was used to carry out this classification. The physical parameters of both groups of fruits were evaluated. Subsequently, a total of 100 elephant apple fruits were collected to evaluate the performance of the developed ACC in comparison to TCM.

### 2.2. Traditional Cutting Method

Traditionally, elephant apples are manually cut using a knife or other traditional tools, as depicted in Figure 1B. However, the conventional tools currently in use suffer from poor design, contributing to the prevalence of various work-related musculoskeletal disorders (MSDs). The prevalent method involves squatting or sitting on the floor with folded knees, a common practice during fruit cutting. In this position, the body assumes a folded configuration, with the buttocks and thighs lacking support, potentially leading to musculoskeletal discomfort. Moreover, the use of conventional tools often results in the misalignment of the body’s orientation, exacerbating discomfort. Both sitting and squatting while employing traditional tools frequently induce a range of discomforts, including back pain [1].

### 2.3. Foot-Operated Core Cutter

The FOCC comprises a supporting frame, core cutter, fruit holder, collecting boxes, connecting links, extension spring, and a foot press lever (Figure 2). The force distribution foot press lever is linked to an adjacent shaft, connecting to two other shafts through links, one of which is attached directly to the movable fruit holder. Positioned at the top center of the frame, the immovable core cutter can be removed if necessary. Two collecting boxes, one below the fruit holder and one above the core cutter, are mounted directly to the frame for gathering sepals and fruit cores separately.

Upon pressing the foot lever, compressive forces act on the connected shafts, causing the fruit holder to ascend and bore into the core cutter. Disengaging the foot press lever causes the fruit holder and shafts to return to their original positions due to tension from the extension spring. As the fruit holder descends, sepals collect in the box below, and while the core may adhere to the cutter die, it is easily dislodged by the pressure generated during subsequent fruit cutting. The separated fruit cores accumulate in the upper collecting box positioned above the core cutter [1].

### 2.4. Separation of the Central Core

One of the crucial postharvest processes in the processing of elephant apples is the removal of the pulp/central core. The main aim of this study is to overcome the problems faced while removing the unusable portion of the fruit. To contribute to the effective design of the machine, the central core was separated and its physical characteristics were investigated along with the whole fruit.

### 2.5. Analysis of Physical Properties of Elephant Apple Fruit and Central Core

The design of equipment for various postharvest processing procedures, such as cleaning, sorting, grading, size, cutting/coring, packaging, transporting, and storing, is heavily influenced by the physical characteristics of fruits and their unusable part (pulp). Various physical properties such as dimensions, shape, arithmetic mean diameter (D_a_), geometric mean diameter (D_g_), surface area (S), sphericity (∅), aspect ratio (R_a_), fruit mass, fruit density (ρf), percentage edible matter, percentage non-edible matter, sepal thickness, moisture content, etc., were determined as described below.

#### 2.5.1. Dimensions

Fifty fruits from both groups (groups 1 and 2) were selected for evaluation of size and shape. A vernier caliper (Kanon Instrument, Tokyo, Japan) with a 0.01 mm precision was used to measure the dimensions: length (L), width (W), and thickness (T). The central cores of the respective fruits were subjected to further measurement. The shape of the elephant apple fruit such as symmetry, uniformity, smoothness, and irregularities were assessed through visual inspection.

#### 2.5.2. Arithmetic Mean Diameter

The arithmetic mean diameter (D_a_) of elephant apple fruit was determined using primary dimensions. Equation (1) was used to determine the arithmetic mean diameter of the fruit [10,11]:

(1) $$D_{a}=\left[\frac{L+W+T}{3}\right]$$

#### 2.5.3. Geometric Mean Diameter

The geometric mean diameter (D_g_) of the elephant apple fruit was determined based on primary dimensions. Equation (2) was used to determine the geometric mean diameter of large fruits and vegetables [10,11]:

(2) $$D_{g}=\left[{\left(L\times W\times T\right)}^{\frac{1}{3}}\right]$$

#### 2.5.4. Surface Area

The surface area (S) of the fruit was determined using Equation (3) according to Mohsenin [12]:

(3) $$S=\left[\pi D_{g}^{2}\right]$$

#### 2.5.5. Sphericity

The sphericity index (∅) of the fruit was determined using the standard method [10,13]. Equation (4) was used to determine the sphericity of elephant apple fruit:

(4) $$Sphericity\left(\emptyset \right)=\left[\frac{D_{g}}{L}\right]$$

#### 2.5.6. Aspect Ratio

The aspect ratio (R_a_) of the fruit was expressed by using Equation (5) according to Mohsenin [12]:

(5) $$Aspectratio\left(R_{a}\right)=\left[\left(\frac{W}{L}\right)\times 100\right]$$

#### 2.5.7. Weight

The weight of each fruit was weighed using an electronic balance (Denver, CO, USA) of range 0–5 kg (least count 1 g) to an accuracy of 0.001 g.

#### 2.5.8. Density

The true volume of the fruit was determined using Archimedes’ principle. Elephant apple fruits of known weight were suspended from the arm of a scale and successively immersed in a container filled with water of known density. The change in weight of the fruits when immersed in water was recorded for each fruit [14]. The true density was then calculated using Archimedes’ principle, which states that the buoyant force on an object immersed in a fluid is equal to the weight of the fluid displaced by the object. The density (ρ) of the fruit was calculated using Equation (6):

(6) $$\rho =\left[\frac{m}{V}\right]$$

where ρ is fruit density, m is the mass of fruit and V is the volume of water displaced.

#### 2.5.9. Percentage Edible Matter

The percentage edible content of elephant apple fruit was determined by weighing the whole sample before removing the non-usable part (core or pulp) [15]. Equation (7) was used to calculate the percentage of edible matter for elephant apple fruit.

(7) $$Edible\;matter(\%)=\left[\left(\frac{Wt.\;of\;consumable\;matter\;(kg)}{Wt.\;of\;whole\;fruit\;(kg)}\right)\times 100\right]$$

#### 2.5.10. Percentage Non-Edible Matter

The percentage of non-edible matter in elephant apple fruit mainly consists of the central core (pulp). Equation (8) was used to determine the percentage of non-edible matter in the elephant apple fruit [15].

(8) $$Non-edible\;matter\;(\%)=\left[\left(\frac{Wt.\;of\;non-consumable\;matter\;(kg)}{Wt.\;of\;whole\;fruit\;(kg)}\right)\times 100\right]$$

#### 2.5.11. Sepal Thickness

The thickness of the sepal is considered to be an important characteristic. To determine the thickness of the sepals of elephant apple fruit, the first step was to excoriate the sepals from the fruit. The thickness of the sepals was then measured with the help of the vernier caliper [15].

#### 2.5.12. Moisture Content

The sample’s moisture content was estimated using Sartorius MA 160 Moisture Analyzer. Initially, the disposable aluminium pan is loaded blank in the moisture analyzer for calibration. After calibration was completed, the fruit sample was transferred into the aluminium pan and the hood was closed for the moisture analyzer to run the test at 105 °C. Following completion of the test after 5–10 min, the moisture analyzer stops automatically and displays the percentage of moisture content of the tested sample. Subsequently, the moisture content was also evaluated using the oven drying method recommended by AOAC [16].

### 2.6. Design Development

**Figure 3:**
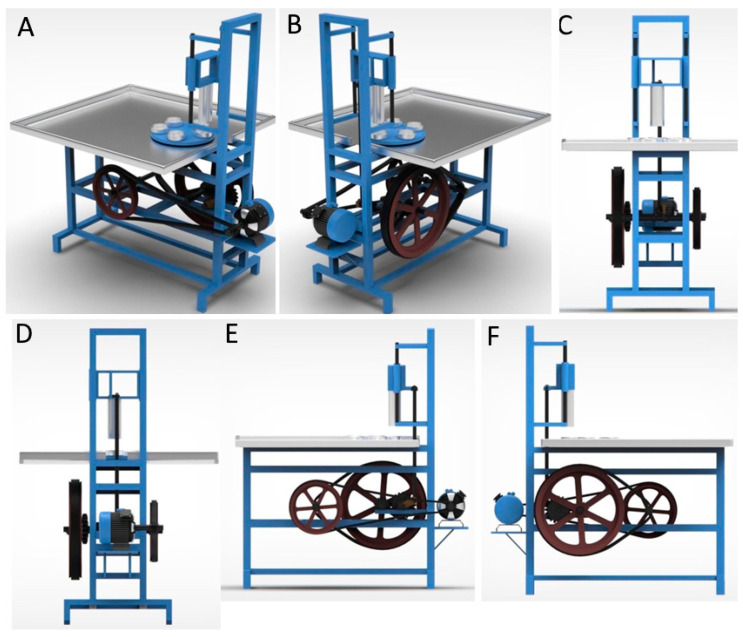
Three-dimensional design of automatic core cutter with different orientations: (**A**,**B**) perspective view, (**C**) front side view, (**D**) back side view, and (**E**,**F**) left- and right-side view.

The 3D model of the ACC was developed using Autodesk Maya 2023.2 software. Figure 3 depicts the three-dimensional design and its orientations.

### 2.7. Fabrication

The material acquisition process is crucial in the manufacturing of a new machine to meet operational and hygienic standards. Elephant apple fruits are acidic and can rapidly oxidize mild steel (MS) resulting in the transfer of hazardous elements into the processed fruit. Thus, it was ensured that parts of ACC (core cutter, circular disc with fruit holders, and collecting tray) coming in contact with the sample were made of stainless steel (SS) and the remaining components involved in operation and frame were built with MS. The raw materials used in the manufacturing of the ACC and its associated costs are provided in Table 1, and the measurements and technical details of the parts and components are provided in Table 2.

The entire fabrication process consists of labelling, carving, welding, grinding, polishing, and painting. The tee and corner joints were welded using an electric arc, the cutter die was sharpened, and the joints were smoothed with an angle grinder. The cost of fabrication of the ACC includes the raw materials and fabrication costs [17]. Fabrication of the cutter costs nearly USD 663.88 (INR 55,000).

### 2.8. Parts of the ACC

**Figure 4:**
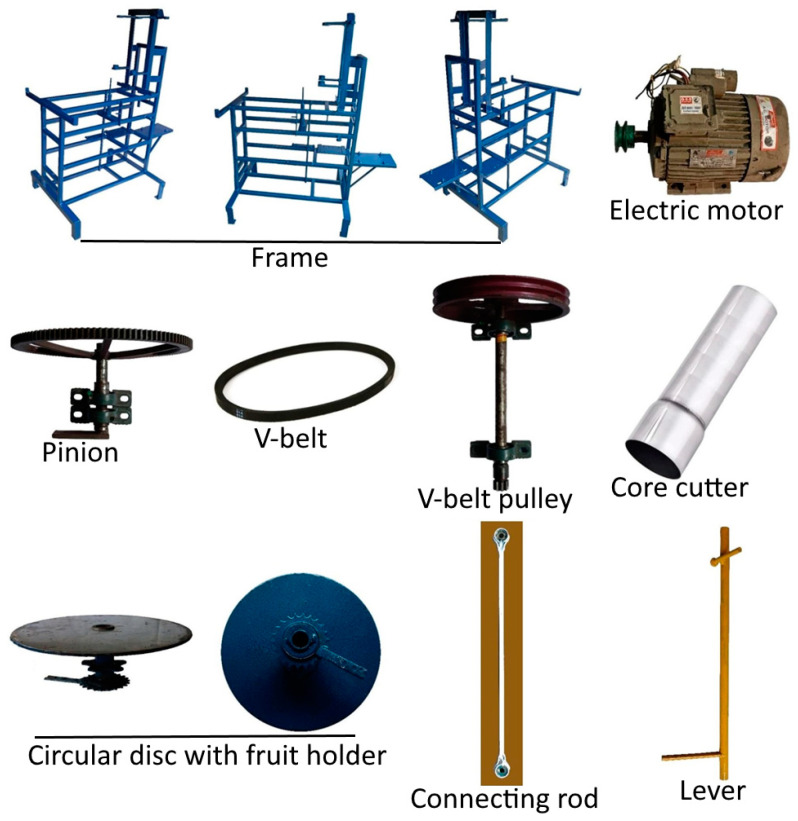
Frame and components used while fabrication of automatic core cutter.

The frame parts and various ACC components used during fabrication are provided in Figure 4. The main parts, namely the circular disc with fruit holders and core cutter, have been covered in this section.

#### 2.8.1. Core Cutter

The most significant part of ACC is the core cutter. It was designed to remove the central core of elephant apple fruit densely coated with closely adhering imbricate sepals. The movable core cutter is a round SS tube of cutting edge on one side. The replaceable core cutter is 20 cm long and has a variable diameter of 5, 6, or 7 cm depending on the size of the fruit. To determine the size of the core cutter, the physical characteristics of the fruit and its central core were considered [9].

#### 2.8.2. Fruit Holder

The fruit holders attached to the circular disc fulfil the function of firmly keeping the fruit in place while the disc rotates and stops at the center where the core cutter is positioned. The disc with fruit holders rotates when the core cutter moves upwards and stops when the cutter moves downwards for the coring of the fruit. An assembly has been made between the movement of the cutter and the rotation of the disc using different links, connecting springs and gears for the smooth operation of the disc.

### 2.9. Operating Principle

The ACC consists of a supporting frame, core cutter, circular disc with five fruit holders, lever, collecting tray, connecting links, extension spring, connecting rod, connecting shafts, bearings, electric motor, v-belt pulley, crankshaft, motor pulley, and pinion gear. A force distribution 745.7 W electric motor drives the v-belt pulley mounted on the right side of the frame using a v-belt. The v-belt pulley simultaneously rotates the pinion gear mounted on the left side of the frame by means of a shaft. The shaft connecting the v-belt pulley and pinion gear is supported by two bearings attached directly to the frame. The pinion gear drives the gearbox assembly mounted below the frame which then transmits force by converting rotational motion into reciprocating motion via the crankshaft to the connecting rod linked to the movable core cutter for coring the fruit and releasing back to its original position with every two rotations.

Simultaneously, when the cutter moves upwards it drives the lever which is connected to the base of the circular disc (with fruit holders) by means of flat iron bars and connecting springs which help to stop or release the rotation of the disc, allowing the next fruit holder to maintain its position at the center of the cutter. A collecting tray is directly attached to the frame, beneath the circular disc for collecting the fruits’ sepals and central core. The core might adhere to the die of the cutter throughout this operation, but it can be removed with little effort owing to the force exerted while cutting/coring the other fruits consecutively. The whole operation is continuous and after the core of the fruit is removed the sepals can easily be separated for further processing.

### 2.10. Performance Metrics

Performance metrics like cutter capacity and sepal yield for both ACC and TCM were assessed using experimental trials for 5 batches of fruit, each consisting of 10 elephant apples. The systematic approach for assessing each performance metric is discussed in the section below.

#### 2.10.1. Cutter Capacity

The machine’s capacity was determined using Equation (9) according to Behera and Rayaguru [18].

(9) $$Q=\left[\frac{W}{T}\times 60\right]$$

where,

Q = ACC Capacity, kg/h,
W = Total weight of processed fruit, kg,
T = Time, min.

#### 2.10.2. Sepal Yield

Sepal yield was determined using Equation (10) according to Saikia et al. [1].

(10) $$Y=\left[\frac{W_{2}}{W_{1}}\times 100\right]$$

where,

Y = Sepal yield, %,
W_1_ = Total weight of processed fruit, kg,
W_2_ = Total weight of obtained sepals, kg.

### 2.11. Cost Analysis

The cost analysis for the fabricated machine was conducted in accordance with the guidelines provided by the Ministry of Micro, Small and Medium Enterprises (MSME), Government of India, and the Food and Agriculture Organization (FAO). Various parameters viz. Benefit-Cost Ratio (BCR), Pay-Back-Period (PBP) and Return-On-Investment (ROI) were calculated by standard procedure given in Equations (11)–(13). For the developed machine, fixed costs, variable costs, revenue collected, and annual profit were accounted for [19,20].

(11) $$BCR=\left[\frac{Annual\;net\;profit}{Annual\;Fixed\;cost+Annual\;Variable\;cost}\right]$$

(12) $$ROI(\%)=\left[\left(\frac{Total\;annual\;revenue-Annual\;present\;value\;of\;fruit}{Annual\;Variable\;cost}\right)\times 100\right]$$

(13) $$PBP=\left[\frac{1}{BCR}\right]$$

The Pay-Back-Period (PBP) is the time needed to recover the money invested or to achieve the break-even point. ROI represents the relationship between the profit or loss generated within a year in relation to an investment, depicted as a percentage indicating the rise or fall in the investment’s value during that specific year [19]. The cost of processing 1 kg of elephant apple fruit can be calculated by dividing the total annual operating cost, excluding the cost of elephant apple fruit, by the annual processing capacity of the fruits.

### 2.12. Statistical Analysis

The experiments were performed in triplicate and the results were presented as the mean value along with the standard deviation (SD). To determine any significant differences between groups, Duncan’s multiple range test was applied using one-way ANOVA (*p* < 0.05) with the help of IBM^®^ SPSS^®^ Statistics version 22.0 (IBM Ltd., Armonk, NY, USA) and Microsoft Excel^®^ version 2019 (Microsoft Co., Ltd., Redmond, WA, USA).

## 3. Results and Discussion

**Figure 5:**
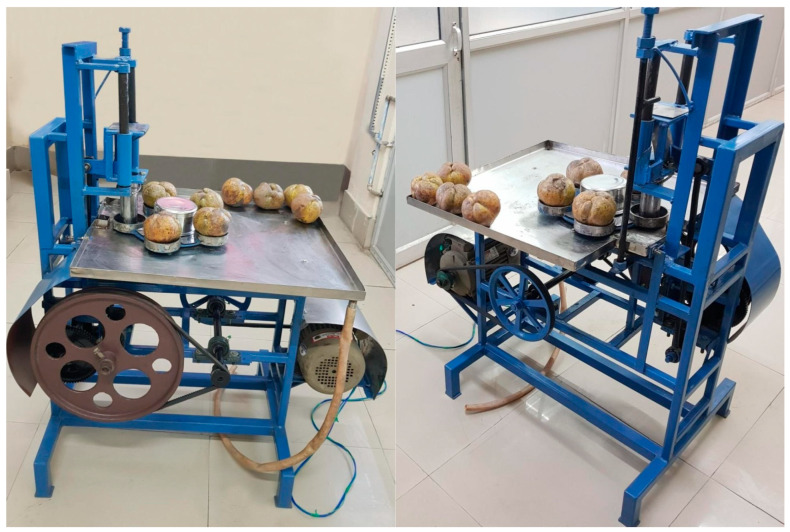
Developed automatic core cutter.

The physical characteristics of the fruit and central core as well as operational standards were used to establish the measurements of the ACC and its components. The fabricated cutter was put to the test, as pictured in Figure 5, and its performance was evaluated together with cost analysis.

### 3.1. Physical Properties of Elephant Apple Fruit and Central Core

The physical properties of the elephant apple fruit and its central core for both the groups (groups 1 and 2) were investigated and the findings have been presented in Table 3. The main goal of this investigation is to enhance the separation of the fruit’s core while reducing sepal loss. Therefore, finding out the physical properties of the central core is also essential for designing and building the cutter.

### 3.2. Performance Evaluation

Investigations were carried out to see how effectively the ACC performed in comparison to the TCM and FOCC for cutting/coring of elephant apple for 5 batches, each of size 10. From Table 4, it is noted that compared to the FOCC reported by Saikia et al. [1] and the TCM, the ACC was found to be approximately 18.89 times faster than the TCM and 2.43 times faster than the FOCC. For 5 batches each of size 10, the TCM and FOCC required 1133.40 ± 58.19 and 145.80 ± 1.30 s to cut, while the ACC took just 60 ± 0 s. A negligible deviation in cutting/coring time means no change in cutting/coring capacity for the ACC whereas a higher deviation in TCM can be attributed to the discrepancy in cutting time taken by the individual. Compared to the TCM (12–15 kg/h) and FOCC (115–130 kg/h), the ACC has a higher capacity (270–300 kg/h) of processing elephant apples. The results demonstrate that TCM is not merely time-consuming but also unreliable [1].

### 3.3. Cost Analysis of the ACC

Proper cost-economic analysis is essential for validating the technology and commercializing the designed machine. The costs associated with obtaining sepals and operating the machine are directly influenced by the raw materials used. For this study, it was assumed that elephant apple fruits were gathered during the peak harvest season (September to January), when their price is typically at its lowest. The average procurement cost of elephant apple fruits was 3.50 INR/kg. The analysis considered 150 operation days per year, with each day consisting of 7 h of operation. Machine depreciation over a five-year period was factored in, along with an annual maintenance cost of 2% of the machine’s initial cost. Additionally, insurance cost (2% of the machine cost), interest on capital (10% per annum), and daily labor wages (@ INR 350) were accounted for [20]. Sepals obtained from processed/cleaned elephant apples have a market price of 15 INR/kg. Further details regarding the cost analysis are shown in Table 5 and compared with the FOCC discussed by Saikia et al. [1].

The estimated amounts for revenue collected, fixed costs, and variable costs, as well as the profit generated annually, are presented in Table 5. According to the study, the ACC can generate a net annual profit of INR 2,347,965, with a BCR of 1.85:1, ROI of 197.34%, with a PBP of 0.54 yr compared to the FOCC [1] generating a net annual profit of INR 947,771, with a BCR of 1.60:1, ROI of 178.24%, with a PBP of 0.63 yr. The cost of processing 1 kg of elephant apple fruit for the ACC was found to be INR 0.92 compared to the FOCC [1] with INR 1.40. This shows that the ACC would clearly cater to the needs of growers and small business owners for postharvest processing of sizable elephant apple fruits at affordable prices and thus, might be a viable business prospect for the low-income population of regions where elephant apple is abundantly found. Moreover, setting up a small enterprise for postharvest processing of elephant apples will create 300 man-days of job opportunities in rural areas.

## 4. Conclusions

Elephant apples contain bioactive compounds with medicinal properties and have been traditionally consumed by native people in regions where they are abundant. However, their large-scale utilization is hindered by challenges in postharvest processing, such as cutting and coring. To address this issue, an automatic elephant apple core cutter (ACC) was engineered to reduce workplace health risks and improve job effectiveness during the cutting process. The overall analysis of experimental results demonstrated that the ACC outperformed the TCM and FOCC in terms of performance. Moreover, the ACC developed is found to be significantly improved in every aspect compared to the TCM, from operation to handling and from sepal recovery to cutting/coring capacity. In terms of operation and handling, the ACC only requires labor for placing the fruits in the fruit holders, without the requirement of any extra physical effort compared to the laborious tasks involved in TCM. In terms of recovery, the ACC provides a greater sepal yield of 85.68 ± 1.80% and more consistent coring of the elephant apple than the TCM, with 64.23 ± 2.43% sepal yield. In terms of performance, the ACC can process 270–300 kg/h compared to the FOCC with 115–130 kg/h and the TCM with only 13–17 kg/h. The developed cutter can generate a net annual profit of INR 2,347,965 with a BCR of 1.85:1, ROI of 197.34%, and a PBP of 0.54 yr. This shows that the ACC would evidently cater to the needs of farmers and small-scale business owners for large-scale postharvest processing of elephant apple at reasonable rates and therefore may be a good business proposal for the low-income population of regions where elephant apple is widely found, including India.

## Figures and Tables

**Table 1:** Raw materials used in the manufacturing of the automatic core cutter and its associated costs ^1^.

Details of Items	Quantity (kg)	Cost Unit (USD)	Total Cost (USD)
Stainless steel sheet 316	15	5.43	81.48
Mild Steel Angle, Flat Bar, Sheet, Rod, Pipe, Plate, Channel, Square Pipe, etc.	120	1.21	144.85
Power Transmission: Bearings, Pulley, Pinion, V-Belt, Shaft, etc.			117.69
Fastening Components: Nuts, Bolts, Screws, Springs, Fly Nuts, Washers, etc.			12.07
Oil and Lubricants, Grease, etc.			6.04
Power Unit (Electric Motor, Electric Wire, Cable, Switches, etc.)			181.06
Metallic Paint			24.14
Fabrication Cost			96.56
Total Cost			663.88

^1^ Cost of raw materials (2024) used to design and build a single cutter, which may be expected to vary with time and large-scale manufacturing. The cost is expected to vary with time and large-scale manufacturing. USD, United States Dollar; 1 INR (Indian rupee) = USD 0.012 as of March, 2024.

**Table 2:** Dimensions and specifications of components used in the fabrication of automatic core cutter.

Component of Cutter	Material Type	Dimensions
Frame (L × B × H)	Mild steel	1067 × 610 × 1270 mm
Disc with fruit holder	Stainless steel 316	305 mm (Diameter)
Fruit holder	Stainless steel 316	80 mm (Diameter) × 5
Lever	Mild steel	550 mm
Collecting Tray (L × B)	Stainless steel 316	990 × 610 mm
Cutter die	Stainless steel pipe 316	50 mm (Diameter)
Connecting Shafts	Mild steel pipe	300 and 200 mm
Connecting Rod	Mild steel pipe	500 mm
V-belt Pulley	Mild steel	305 mm (Diameter)
V-belt	Synthetic Rubber	1372 mm
Pinion Gear	Case Carburized Steel	381 mm (Diameter)
Motor Pulley	Mild steel	76 mm (Diameter)
Bearing	Steel	7 (Pcs)
Electric Motor	-	745.7 W

**Table 3:** Physical properties of whole fruit and central core.

Physical Properties	Group 1		Group 2	
	Whole Fruit	Central Core	Whole Fruit	Central Core
Length (L), mm	116.85 ± 5.45	68.43 ± 4.22	94.41 ± 3.69	54.26 ± 3.67
Width (W), mm	103.44 ± 6.72	59.64 ± 4.37	82.93 ± 5.37	45.19 ± 3.54
Thickness (T), mm	102.21 ± 5.24	57.81 ± 3.73	81.26 ± 4.24	44.36 ± 3.15
Arithmetic mean diameter (D_a_), mm	107.50 ± 5.80	61.96 ± 4.11	86.20 ± 4.43	47.94 ± 3.45
Geometric mean diameter (D_g_), mm	107.30 ± 5.77	61.79 ± 4.09	86.01 ± 4.38	47.74 ± 3.45
Surface area (S), mm^2^	36,151.73 ± 104.54	11,988.53 ± 52.53	23,228.84 ± 60.24	7156.40 ± 37.37
Sphericity (∅)	0.92 ± 0.05	0.90 ± 0.06	0.91 ± 0.05	0.88 ± 0.06
Aspect ratio (R_a_)	0.89 ± 0.06	0.87 ± 0.06	0.88 ± 0.06	0.83 ± 0.07
Weight, g	475.84 ± 4.18	90.79 ± 5.16	432.36 ± 5.35	78.74 ± 6.71
Density, g/cm^3^	0.99 ± 0.02	-	0.99 ± 0.02	-
% edible matter	79.45 ± 5.53	-	80.13 ± 6.12	-
% non-edible matter	19.40 ± 4.25	-	18.69 ± 5.52	-
Sepal thickness, mm	6.62 ± 1.34	-	4.84 ± 1.18	-
Moisture content, % w.b.	87.57 ± 1.59	-	85.93 ± 2.06	-

**Table 4:** Performance evaluation of automatic core cutter compared to foot-operated elephant apple core cutter and traditional cutting method.

Batch (10 Elephant Apples Each)	Traditional Cutting Method					Foot-Operated Core Cutter ^1^					Automatic Core Cutter				
	Cutting Time (s)	Mass of Fruit (g)	Mass of Sepals (g)	Sepal Yield (%)	Mass of Pulp (g)	Cutting Time (s)	Mass of Fruit (g)	Mass of Sepals (g)	Sepal Yield (%)	Mass of Pulp (g)	Cutting Time (s)	Mass of Fruit (g)	Mass of Sepals (g)	Sepal Yield (%)	Mass of Pulp (g)
1	1036	4389	2832	64.53	1466	147	4371	3680	84.18	667	60	4518	3973	87.94	512
2	1164	4471	2938	65.71	1454	145	4748	4118	86.73	611	60	4597	3827	83.25	741
3	1187	4632	2980	64.33	1568	144	4692	3911	83.35	749	60	4641	4019	86.59	589
4	1148	4716	3151	66.82	1487	146	4763	4079	85.64	658	60	4483	3795	84.65	653
5	1132	4538	3058	67.39	1409	147	4574	4016	87.80	536	60	4821	4145	85.98	638
Total	5667	22,746	14,959	-	7384	729	23,148	19,804	-	3221	300	23,060	19,759	-	3133
Mean	1133.4	4549.2	2991.8	65.76	1476.8	145.8	4629.6	3960.8	85.55	644.2	60	4612	3951.8	85.68	626.6
SD	58.19	129.02	120.72	1.35	58.43	1.30	162.54	175.40	1.81	78.27	0	132.5	143.57	1.8	84.35

^1^ The information is reproduced from Saikia et al. [1] and is an open access article (copyright © 2024 by authors under exclusive license to Springer Nature Switzerland AG) distributed under the terms and conditions of the Creative Commons Attribution (CC BY) license. In this investigation the sepals obtained and the pulp which holds minor portions of sepals together represents the total recovery. The total recovery for ACC was found to be 99.27% with 0.73% tangible loss whereas TCM implicated a higher tangible loss of 1.77% with a total recovery of 98.22%. This higher tangible loss can be attributed to the release of viscous fluids when the elephant apple’s sepals were ruptured due to improper cutting of the fruit. The ACC yielded 85.68 ± 1.80% of sepals, whereas the TCM leads to a greater loss of usable fruit sections yielding only 65.76 ± 1.35% of sepals from the total mass of fruits processed. The specifically designed ACC is targeted towards uniform coring of the fruit with less wastage of the usable sections of fruit. Since this aspect is crucial for the industrial applications of the product obtained, the ACC may add considerably to postharvest processing of elephant apple fruits with undamaged sepals. The results obtained by ACC are consistent with those obtained by FOCC [1].

**Table 5:** Cost economic analysis for the developed automatic core cutter ^1^.

Parameters	Values	
	FOCC	ACC
Machine Parameters		
Cutter Cost, INR	3000	55,000
Effective capacity, kg of fruit processed/h	115	270
Life of cutter, year	3	5
Operating hrs/day	7	7
Operating days/year	150	150
Electricity consumption at 0.746 kWh, units per year	-	784
Fixed Cost		
Annual processing capacity of the raw material (elephant apple fruit), kg	120,750	283,500
Depreciation of the machine (salvage value @ 10% of initial cost), INR	300	5500
Interest on cutter cost @ 10% p.a., INR	300	5500
Insurance @ 2% p.a. of cutter cost, INR	60	1100
Housing @ 2% of the initial cutter cost/year, INR	60	1100
Annual Fixed Cost, INR	720	13,200
Variable Cost		
Maintenance cost annually @ 2% of cutter cost, INR	60	1100
Wages of two labor annually @ 350 INR per labor per day	105,000	105,000
Annual electricity charge @ 8.00 INR/kWh	-	6272
Cost of elephant apple fruit @ 3.50 INR/kg	422,625	992,250
Interest @ 10% p.a., INR on cost of procured elephant apple fruit	42,263	99,225
Cost of storage/losses of elephant apple fruit annually @ 5% of its cost, INR	21,131	49,613
Annual Present Value of Elephant Apple Fruit, INR	486,019	1,141,088
Annual Operating/Variable Cost, INR	591,079	1,253,460
Revenue		
Average content of elephant apple sepals in fruit, %	85	85
Annual production of edible elephant apple sepals, kg	102,638	240,975
Annual production of unused central core per kg of elephant apple, kg	18,112	42,525
Total Annual Revenue for Sale of Elephant Apple Sepals @ 15.00 INR/kg, INR	1,539,570	3,614,625
Annual Net Profit (Total Annual Revenue—(Fixed Cost + Variable Cost)), INR	947,771	2,347,965
Benefit-Cost Ratio (BCR)	1.60	1.85
Return-On-Investment (ROI, %)	178.24	197.34
Pay Back-Period (PBP, Year)	0.63	0.54
Cost of processing 1 kg of elephant apple fruit, INR	1.40	0.92

^1^ INR, Indian Rupees; 1 USD = 82.85 INR as of March 2024. ACC, automatic core cutter; FOCC, foot-operated core cutter.

## Data Availability

The original contributions presented in the study are included in the article, further inquiries can be directed to the corresponding authors.
